# Mastectomy or Breast-Conserving Therapy for Early Breast Cancer in Real-Life Clinical Practice: Outcome Comparison of 7565 Cases

**DOI:** 10.3390/cancers11020160

**Published:** 2019-01-31

**Authors:** Stefanie Corradini, Daniel Reitz, Montserrat Pazos, Stephan Schönecker, Michael Braun, Nadia Harbeck, Christiane Matuschek, Edwin Bölke, Ute Ganswindt, Filippo Alongi, Maximilian Niyazi, Claus Belka

**Affiliations:** 1 Department of Radiation Oncology, LMU University, 81377 Munich, Germany; daniel.reitz@med.uni-muenchen.de (D.R.); montserrat.pazos@med.uni-muenchen.de (M.P.); stephan.schoenecker@med.uni-muenchen.de (S.S.); maximilian.niyazi@med.uni-muenchen.de (M.N.); claus.belka@med.uni-muenchen.de (C.B.); 2 Department of Gynecology and Obstetrics, Red Cross Hospital, 80637 Munich, Germany; michael.braun@swmbrk.de; 3 Breast Center, Department of Gynecology and Obstetrics, LMU University, 81377 Munich, Germany; nadia.harbeck@med.uni-muenchen.de; 4 Department of Radiation Oncology, Heinrich Heine University, Medical faculty, 40225 Düsseldorf, Germany; matuschek@med.uni-duesseldorf.de (C.M.); boelke@med.uni-duesseldorf.de (E.B.); 5 Department of Radiation Oncology, Medical University, 6020 Innsbruck, Austria; ute.ganswindt@i-med.ac.at; 6 Department of Radiation Oncology, Sacro Cuore Don Calabria Hospital, 37024 Negrar-Verona, University of Brescia, 25121 Brescia, Italy; filippo.alongi@sacrocuore.it

**Keywords:** breast cancer, breast-conserving therapy, mastectomy, outcome, comparative effectiveness

## Abstract

Although the organ preservation strategy by breast-conserving surgery (BCS) followed by radiation therapy (BCT) has revolutionized the treatment approach of early stage breast cancer (BC), the choice between treatment options in this setting can still vary according to patient preferences. The aim of the present study was to compare the oncological outcome of mastectomy versus breast-conserving therapy in patients treated in a modern clinical setting outside of clinical trials. 7565 women diagnosed with early invasive BC (pT1/2pN0/1) between 1998 and 2014 were included in this study (median follow-up: 95.2 months). In order to reduce selection bias and confounding, a subgroup analysis of a matched 1:1 case-control cohort consisting of 1802 patients was performed (median follow-up 109.4 months). After adjusting for age, tumor characteristics and therapies, multivariable analysis for local recurrence-free survival identified BCT as an independent predictor for improved local control (hazard ratio [HR]:1.517; 95%confidence interval:1.092–2.108, *p* = 0.013) as compared to mastectomy alone in the matched cohort. Ten-year cumulative incidence (CI) of lymph node recurrences was 2.0% following BCT, compared to 5.8% in patients receiving mastectomy (*p* < 0.001). Similarly, 10-year distant-metastasis-free survival (89.4% vs. 85.5%, *p* = 0.013) was impaired in patients undergoing mastectomy alone. This translated into improved survival in patients treated with BCT (10-year overall survival (OS) estimates 85.3% vs. 79.3%, *p* < 0.001), which was also significant on multivariable analysis (*p* = 0.011). In conclusion, the present study showed that patients treated with BCS followed by radiotherapy had an improved outcome compared to radical mastectomy alone. Specifically, local control, distant control, and overall survival were significantly better using the conservative approach. Thus, as a result of the present study, physicians should encourage patients to receive BCS with radiotherapy rather than mastectomy, whenever it is medically feasible and appropriate.

## 1. Introduction

In the early 1980s, large randomized studies first proved that breast-conserving surgery (BCS) followed by postoperative radiotherapy (breast-conserving therapy, BCT) was a valid therapeutic alternative to radical mastectomy in women with early breast cancer (BC) [1,2]. Nowadays, in this setting, breast organ preservation by BCT has become the treatment of choice, due to the excellent outcome and optimal tolerability. Nevertheless, various population-based studies showed that mastectomy is still considered a concrete treatment option and continues to be chosen by several patients with BC in daily clinical practice [3,4,5,6].

Treatment of early-stage breast cancer can be considered as a preference-sensitive setting, where decision-making between treatment options can change according to patient preferences [7]. Typical factors able to influence the therapeutic choice in favor of mastectomy include: (i) concerns regarding cancer recurrence, (ii) perception that health outweighs breast retention [8], or (iii) perceived consequences of BCT, including potential adverse effects of radiation therapy [7,9]. Moreover, a renewed interest and trend towards mastectomy has recently emerged, with an increased use of skin-sparing or nipple-sparing mastectomies with immediate breast reconstruction [10,11,12]. This treatment strategy provides superior aesthetic and quality-of-life outcomes when compared to radical mastectomy. Nevertheless, long-term oncologic outcomes of these new surgical approaches are currently not provided and only retrospective studies were available as evidence. Moreover, the demand for more radical surgical therapies has recently gained wide public attention, as prophylactic mastectomy in BRCA gene mutation positive celebrities attracted notable media interest [13]. The prominence of this issue in the media might also have influenced oncologic patient´s preferences regarding their choice of surgical management. In fact, shared decision-making in daily clinical practice is strongly influenced by a number of confounding factors, including clinician preferences and trade-offs regarding toxicity risks or comorbidities [14].

BC management has changed dramatically over time, and local recurrence rates after BCT have decreased significantly [15]. The impact of mammography screening in downward stage migration resulted in smaller tumor sizes and less extensive nodal involvement and is accompanied by improvements in adjuvant treatments, tailored to disease biology. 

Therefore, the aim of the present study was to compare the oncological outcome of mastectomy versus breast-conserving therapy in patients treated in “real life”, in a modern clinical setting outside of clinical trials. 

## 2. Results

### 2.1. Baseline Patient Characteristics

The final study cohort consisted of 7565 women and the subgroup analysis of the matched cohort included 1802 patients. Patient and treatment characteristics for both cohorts are summarized in Table 1. Overall, 84.8% (6412/7565) of patients were treated with BCS and postoperative RT, while 15.2% (1153/7565) a received mastectomy. A significant decrease of mastectomy was documented over time. While in 1998 approximately 21% of patients received a mastectomy, the proportion decreased to 12% in 2014. 

Patients treated with BCT were significantly younger, with a median age at diagnosis of 58.2 years in the BCT group, as compared to 59.3 years in the mastectomy group (*p* < 0.001). Furthermore, patients treated with mastectomy presented with more high-risk features such as tumor size ≥20 mm (43.1%), positive lymph nodes (31.4%), high tumor grade (27.3%) and negative hormone receptor status (12.0%) than patients receiving BCT. Moreover, mastectomy patients received less adjuvant endocrine therapy (52.0% vs. 43.5%, *p* < 0.001). In the matched cohort, we controlled for all these imbalances. 

### 2.2. Outcome

The median follow-up for the entire cohort was 95.2 months (95%CI: 92.5–97.9) and 109.4 months (95% CI: 104.3–114.5) for the matched cohort. Of the 7565 BC patients, 521 (6.9%) developed local recurrences, 160 (2.1%) lymph node recurrences, and 607 (8.0%) distant metastases.

The cumulative incidence of local recurrence (LR) for the BCT group was 3.2% after 5 years and 8.2% after 10 years. In contrast, the mastectomy group had significantly higher local failure rates with 5.0% 5-year LR and 12.6% 10-year LR rates, respectively (*p* < 0.001, Table 2). 

Multivariable Cox analysis for local recurrence-free survival identified mastectomy (hazard ratio [HR], 1.476; 95% confidence interval [CI], 1.164–1.872, *p* < 0.001) as a significant predictor for local failure. Table 3 summarizes other classic prognostic risk factors that had a significant impact on LR risk at multivariable analysis, including young age <40 years (*p* < 0.001), higher tumor stage (*p* < 0.001), high tumor grade (*p* < 0.001) and negative hormone receptor status (*p* = 0.012). In the matched cohort, type of local treatment, age at diagnosis, and tumors stage confirmed their significant impact on LR risk estimates.

Similarly, lymph node recurrences (LNR) were more frequent in patients undergoing mastectomy only. The cumulative incidence of LNR at 5 and 10 years in the BCT group were 0.9% and 2.2%, respectively, compared to 2.6% and 5.7% in patients receiving mastectomy (*p* < 0.001). This observation was still significant on multivariable analysis. Type of local therapy (mastectomy HR 2.442; 95% CI, 1.675–3.560, *p* < 0.001), higher tumor stage (*p* = 0.006) and high tumor grade (*p* < 0.001) did significantly affect the risk of LNR. Focusing on the impact of the type of local treatment in the matched cohort, mastectomy was also correlated with an increased rate of LNR (HR 1.517; 95% CI, 1.092–2.108, *p* = 0.013, Table 4).

Ten-year distant metastasis-free survival (DMFS) in the entire cohort was statistically different in the univariate analysis—with 90.2% DMFS in the BCT group, compared to 84.8% in the mastectomy group (*p* < 0.001). This was also seen in a comparable magnitude in the matched cohort (*p* = 0.013). Overall, patients treated with postoperative radiotherapy after BCS showed improved distant control, independent from other covariates in multivariable Cox regression analysis (mastectomy HR 1.257; 95% CI, 1.006–1.570, *p* = 0.044). Other factors correlated with poor DMFS in this cohort were advanced tumor stage (*p* < 0.001), high tumor grade (*p* < 0.001) and negative hormone receptor status (*p* = 0.050). Also in the matched cohort, the positive effect of BCT on DMFS was observed (*p* = 0.008, Table 5).

Among patients treated with BCS plus RT, 10-year OS estimates were 86.7%, and for those treated with mastectomy 77.6% (*p* < 0.001). In multivariable Cox regression analysis, the use of mastectomy was again independently associated with less favorable outcome, with an HR of 1.268 (95% CI, 1.055–1.525, *p* = 0.011). Further risk factors correlated with poor OS in this cohort were older age (*p* < 0.001), advanced tumor stage (*p* < 0.001), and high tumor grade (*p* < 0.001). This effect could be confirmed in multivariable analysis for the matched cohort, where type of local treatment (mastectomy HR 1.452; 95% CI, 1.124–1.875, *p* = 0.004, Table 6), older age (*p* < 0.001), advanced tumor stage (*p* < 0.001), and high tumor grade (*p* = 0.033) were independent risk factors.

## 3. Discussion

The present study showed that patients treated with BCS followed by radiotherapy (RT) in a population reflecting “real life” in this clinical setting, had an improved outcome regarding local control, distant control, and overall survival compared to those who underwent a mastectomy. These findings were also confirmed in the matched cohort after adjusting for confounders.

The results presented here are in line with those of other studies investigating the same clinical setting. A population-based analysis of van Maaren et al. [4] of 37,207 breast cancer patients treated in the Netherlands between 01/2000 and 12/2004 obtained similar results. BCS was associated with a significantly improved 10-year overall survival (HR 0.81, 95% CI: 0.78–0.85, *p* < 0.0001). Furthermore, in a representative cohort of patients diagnosed in 2003, BCT had a significant impact on relative survival (HR 0.76, 95% CI: 0.64–0.91, *p* = 0.003). In contrast to the present analysis, distant metastasis-free survival (HR 0.88, 95% CI: 0.77–1.01, *p* = 0.070) was not significantly different in the Dutch cohort, with exception of the T1N0 subgroup (HR 0.60, 95% CI: 0.42–0.85, *p* = 0.004). Yet, in the present analysis, the occurrence of lymph node metastases and distant metastases were both decreased in patients treated with breast-conserving surgery and radiotherapy compared to mastectomy. 

Although locoregional and distant control rates are lacking in most of the published experiences [16,17,18,19], two studies addressed these issues in a similar BC cohort as in the present study [20,21]. An analysis of the prospective Swedish Multicenter Cohort Study including 2767 patients compared BCS with postoperative RT and mastectomy without RT. Similar to the present analysis, the axillary recurrence-free survival rate at 13 years was significantly reduced after mastectomy without irradiation as compared to BCS (98.3% versus 96.2%, *p* < 0.001) [21]. Moreover, locoregional recurrence was a strong independent predictor of breast cancer death, (HR: 4.28, 95% CI: 2.55–7.17) and overall survival (HR: 2.64, 95% CI: 1.66–4.19). 

The axillary recurrence rates decrease after BCS with RT in comparison to mastectomy, which may have different explanations. Of note, in the present study, 23.5% of patients treated in the BCT group had a positive nodal status with 1–3 involved lymph nodes. While after the AMAROS trial [22], the debate about the role of regional nodal irradiation continues, results from the ACOSOG Z0011 trial [23] and the IBCSG 23-01 trial [24] demonstrated that patients with low-volume nodal disease who are treated with BCS and whole breast RT, can safely avoid axillary lymph node dissection without affecting locoregional control or survival rates [25]. The potential rationale behind this observation is that radiation originating from whole-breast tangential field RT after BCS could exert some protective effect on axillary recurrence rates by controlling the minimal residual disease [21]. It is noteworthy, that the dose to axillary lymph node levels I and II usually is significantly lower than the prescribed dose and can range from 5% to 80% of the prescribed dose (mean value 48.7%). Even in patients receiving regional nodal irradiation of 50 Gray (Gy) to the supra-/infraclavicular lymph node levels (corresponding to levels IV, III and interpectoral lymph nodes), level I receives a reduced dose coverage of mean 41.3 Gy [26,27]. The potential influence of whole breast irradiation, especially in cases of pN+, needs further evaluation in randomized studies.

Regarding distant control, in a single center experience of 6137 cases, Wang et al. [20] observed that patients undergoing BCS plus RT showed a significantly increased 5-year metastasis-free survival (*p* < 0.003) and overall survival (*p* < 0.036) compared with mastectomy. But how could these results be interpreted? Is RT able to add something more than just improved locoregional control? The EBCTCG meta-analysis [28] proved a concrete direct relationship between improved local control and favorable breast cancer specific survival outcome. Nevertheless, the underlying biological mechanisms remain unclear. The oncological community has generated various hypotheses regarding the heterogeneous biology of BC and the impact of available treatment options. A commonly accepted hypothesis is that the addition of RT represents an effective curative treatment for a selected subset of patients who would otherwise have relapsed locally and subsequently would have developed metastases. The fact that the survival benefit only occurs in the framework of successful local control, indicates that RT is involved in events occurring within the treated radiotherapy fields. RT prevents local recurrences through the successful eradication of residual tumor clones or tumor cell clusters within the breast, which are not detected at primary diagnosis. Regarding the beneficial effect on distant tumor control, this interpretation assumes that the metastatic process consists of different waves of cell migration and metastases with differing invasive properties [29]. Hence, RT appears to have unique biological effects to prevent early distant dissemination of cancer cells to distant organs. Furthermore, several potential interactions with the immune system are advocated, including radiation-induced tumor-specific immunity capable of rejecting the colonizing clonogenic cells [29]. Nevertheless, it remains challenging to assess the relative contribution of the interactions between systemic and locoregional treatments on the outcome, as well as that of the individual drugs and RT volumes [15].

The present results of patients treated in a “real life” clinical setting are different from those reported from historical randomized trials of the 1980s, which described similar survival for BCT and mastectomy [30,31]. A key to interpreting these different findings could be that the management of breast cancer has changed considerably over time. Fisher et al. [31] documented a 5-year local recurrence rate of 7.7% for the BCT group and 14.3% in the twenty-year follow-up of the NSABP trial B-06. The 5-year and 10-year cumulative incidence of local recurrences in the present analysis were 3.2% and 8.2%, respectively, suggesting improved local control rates with modern breast cancer therapy, even in the setting outside of randomized trials. The modern multimodal treatment approach, including diagnostics, surgery, systemic therapy, and RT procedures, has improved significantly over the last decades and might explain the survival difference in patients treated with breast-conserving surgery plus radiotherapy as compared to patients treated with mastectomy. [15] The 10-year overall survival was significantly improved for patients receiving BCS plus radiotherapy: 86.7% with BCT and 77.6% with mastectomy alone (*p* < 0.001). This difference was also observed in the matched cohort (*p* < 0.001). Improved breast cancer specific and overall survival have been found in several population-based cohorts studies [3,5,32,33,34,35,36,37]. Regarding early-stage breast cancer, Hwang et al. [32] analyzed patients diagnosed with stage I or II breast cancer between 1990 and 2004 and reported improved OS and DSS compared to patients with mastectomy (adjusted hazard ratio for OS entire cohort = 0.81, 95% CI: 0.80–0.83). A registry-based study in Norway also showed comparable results to the present analysis [5]. In multivariate analysis, patients who underwent mastectomy for T1-2/N0-1 BC had an adjusted hazard ratio of 1.65 (95% CI: 1.50–1.82) for OS compared to those who underwent BCT. Similarly, in the present matched cohort, the outcome in terms of OS for patients receiving BCS plus RT was improved (HR: 1.452, 95%CI: 1.124–1.875). Onitilo et al. [35] also compared BCS ± RT versus mastectomy. While overall survival was similar for BCS alone and mastectomy, BCS plus radiation was superior compared to mastectomy alone. The authors concluded, that the survival benefit was not only related to the surgical approach itself but that the addition of adjuvant RT results in a prognostic advantage of BCS plus radiation over mastectomy [35].

We controlled for all variables available in the registry. Unfortunately, we could not account for host-related factors like comorbidities, performance status, or clinician- and patient-related preferences, which may have influenced the clinical treatment decision-making process. It is known that older patients or patients with comorbidities often receive non-standard treatments. A recent analysis of 7581 early stage BC patients diagnosed in 9 European countries analyzed the influence of comorbidities on receiving standard treatments and found that mastectomy was mainly given to elderly women and women with comorbidities [38]. There are several other limitations to effectiveness research due to unpredictable confounding factors and consequently, misinterpretations of treatment and mortality effects should be avoided [39]. In fact, the present observational study may suffer from a “confounding by severity” [40], considering that the severity of the disease (e.g., high-risk factors, tumor biology) could be a potential confounder influencing the indication for mastectomy. Furthermore, survival estimates might be affected by non-tumor-related factors such as age or comorbidities, which could lead to more non-breast cancer deaths. However, we conducted a matched cohort analysis to directly address these concerns and control for these imbalances. Finally, we want to underline that patients of the present study were treated at two specialized breast cancer centers, which in general could have improved outcomes as compared to other settings.

Many previous studies have performed similar analyses with comparable results [4,20,21]. However, a specific characterizing element of the present study is the additional matched case control analysis. This methodology was not used in previously published experiences, which further strengthens the evidence that breast-conserving therapy should be the preferred treatment for patients with early-stage breast cancer when it is medically feasible and appropriate. More specifically, in the present study, we could show that breast-conserving therapy had improved outcome regarding local control, distant control and overall survival as compared to mastectomy alone–even in the matched cohort. Patients were matched regarding a number of variables in order to reduce confounding: 1:1 match for tumor lateralization, tumor size, lymph node status, tumor grade, hormone receptor status, administration of chemotherapy/endocrine therapy and age match with a tolerance of ±2 years for age at diagnosis. This resulted in a cohort, where for each mastectomy patient an exactly matched BET patient with exactly the same tumor formula and treatment history was present. Even in this matched cohort analysis, the effect of the choice of surgical treatment on oncologic outcome remained statistically significant. 

Since many people still believe that mastectomy may be a better choice, we recommend generating more external validity, such as this retrospective study, in order to gain wide public attention. It is well known that there are a number of barriers to compliance with treatment recommendations, including lack of outcome expectancy. If a physician believes that a treatment will not lead to an improved outcome, he is less likely to follow the treatment recommendations. Another explanation for the widespread underuse of treatments that were beneficial in controlled trials could be the lack of consideration of external validity [14]. In general, we hope that the present study contributes to the existing evidence regarding the effectiveness of BCS and radiotherapy in this setting. 

## 4. Materials and Methods 

For the present analysis, all female patients with a first primary unilateral invasive breast cancer diagnosed between 1998 and 2014 and treated at two Breast Cancer Centers (Red Cross Hospital or LMU Munich, München, Germany) were identified. All data were retrieved from the Munich Cancer Registry. The cancer registry routinely collects data on patient’s demographics, primary tumor site, the extent of disease (TNM), histology, treatment, and follow-up. Survival information was obtained systematically through death certificates of health offices. Patients were considered eligible after receiving mastectomy without postoperative radiotherapy (RT) or breast-conserving surgery followed by RT. Patients were excluded if they received neoadjuvant chemotherapy, or in case of histology of ductal carcinoma in situ (*n* = 1.412), lymphoma (*n* = 10) or sarcoma (*n* = 57), or in case of unknown date of initial diagnosis (e.g., tumors from death certificate information only [DCO], *n* = 58). Patients were also excluded if surgery information or pathologic tumor stage was incomplete or missing.

For the present study, women with tumor stages pT1pN0, pT2pN0, pT1pN1 and pT2pN1 (all M0) were selected. For comparison of the standard BCT and mastectomy approaches, we excluded patients with tumor stage ≥pT3 or more than 3 positive lymph nodes (pN2), as postmastectomy RT (PMRT) would have been routinely recommended in these high-risk patients. 

Over the last decades, the use of PMRT was under debate for most intermediate risk patients with small tumor size and limited nodal disease (1–3 positive lymph nodes). Although previous studies provided evidence for a possible survival benefit in intermediate-risk patients [41], PMRT was not uniformly recommended at that time. The standard RT regimen at the Department of Radiation Oncology of the LMU Munich during the observation period was whole-breast irradiation following BCS (50.4 Gy in 28 fractions) using a 3-dimensional conformal tangential field technique with a photon or electron boost of 10–16 Gy to the tumor bed [42]. 

In order to reduce selection bias and confounding, a subgroup analysis of a matched cohort was performed. To compare treatment outcomes within a set of similar patients, 1:1 case-control matching on the following variables was performed: age at diagnosis, tumor lateralization, tumor size, lymph node status, tumor grade, hormone receptor status, administration of chemotherapy and endocrine therapy. The Hormone receptor was defined positive if estrogen and/or progesterone were positive (>1%).

Statistical analyses were conducted using IBM SPSS Statistics version 24.0 (IBM, Amonk, NY, USA). Frequency data were analyzed using the Chi-Square test. Tolerances values of the case-control matching were set to 2 years for age at diagnosis and 0 (exact matches) for all other above-mentioned variables. Cumulative incidence analysis (CI) was used to calculate the time to LR and LNR and the differences were assessed using the Gray’s test for equality of cumulative incidence functions and was performed using R environment for statistical computing and visualization (version. 3.4.0). Distant metastasis-free survival (DMFS) and overall survival (OS) were estimated by the Kaplan-Meier method and tested using the log-rank test. The observation period began after diagnosis of the invasive tumor and ended at the date of distant metastasis occurrence or date of death or the last follow-up for cases without events. In addition, Cox proportional hazards models were used to identify independent prognostic factors related to local recurrence-free survival (LRFS), lymph node recurrence-free survival (LNRFS), DMFS and OS for the different cohorts. The significance level in all analyses was set at 5%. 

## 5. Conclusions

In contrast to the highly selected and homogeneous study populations of randomized trials, this observational analysis included a large patient cohort reflecting “real-life” clinical practice involving a more diverse population, including elderly patients. A fundamental finding of the present study was that patients treated with BCS followed by RT had improved outcome in clinical practice regarding local control, distant control and overall survival as compared to mastectomy alone. Even if randomized trials provide the least biased estimates to compare treatments and remain the gold standard of efficacy research in oncology, observational data should be appreciated when weighing treatment options for breast cancer surgery. As a result of the present study, it seems advisable to continue to encourage future patients to receive BCS with RT rather than mastectomy when it is medically feasible and appropriate.

## Figures and Tables

**Table 1:** Patient and tumor characteristics for the entire cohort and the case control cohort.

Variable	Entire Cohort (*n* = 7565)					Case Control Cohort (*n* = 1802)				
	BCS + RT		Mastectomy		*p*-Value	BCS + RT		Mastectomy		*p*-Value
	n	(%) *	n	(%) *		n	(%) *	n	(%) *	
All	6412	(84.8)	1153	(15.2)		929	(50.0)	929	(50.0)	
Age at diagnosis					<0.001					n.s.
<40 years	353	(5.5)	102	(8.8)		68	(7.5)	62	(6.9)	
40–49 years	1193	(18.6)	221	(19.2)		201	(22.3)	184	(20.4)	
50–59 years	1880	(29.3)	282	(24.5)		241	(26.7)	234	(26.0)	
60–69 years	2043	(31.9)	258	(22.4)		215	(23.9)	225	(25.0)	
>70 years	943	(14.7)	290	(25.2)		176	(19.5)	196	(21.8)	
median (years)	58.2		59.3			58.6		58.8		
Lateralisation					0.007					n.s.
right	3181	(49.6)	522	(45.3)		414	(45.9)	414	(45.9)	
left	3231	(50.4)	631	(54.7)		487	(54.1)	487	(54.1)	
Tumour size					<0.001					n.s.
pT1	4790	(74.7)	656	(56.9)		514	(57.0)	514	(57.0)	
pT2	1622	(25.3)	497	(43.1)		387	(43.0)	387	(43.0)	
Nodal status					<0.001					n.s.
pN0	4904	(76.5)	791	(68.6)		646	(71.7)	646	(71.7)	
pN+ (1–3 LN)	1508	(23.5)	362	(31.4)		255	(28.3)	255	(28.3)	
Tumor stage					<0.001					n.s.
T1N0	3860	(60.2)	492	(42.7)		395	(43.8)	395	(43.8)	
T2N0	1044	(16.3)	299	(25.9)		251	(27.9)	251	(27.9)	
T1N1	930	(14.5)	164	(14.2)		119	(13.2)	119	(13.2)	
T2N1	578	(9.0)	198	(17.2)		136	(15.1)	136	(15.1)	
Resection status					n.s.					n.s.
R0	5769	(98.1)	922	(98.2)		812	(98.5)	740	(98.5)	
R1/R2	112	(1.9)	17	(1.8)		12	(1.5)	11	(1.5)	
[unknown]	531	(8.2)	214	(18.5)		77	(8.5)	150	(16.6)	
Grade					<0.001					n.s.
G1	1248	(19.9)	98	(9.1)		77	(8.5)	77	(8.5)	
G2	3610	(57.6)	684	(63.6)		599	(66.5)	599	(66.5)	
G3/4	1411	(22.5)	294	(27.3)		225	(25.0)	225	(25.0)	
[unknown]	143	[2.3]	77	[6.6]						
Hormone receptor										
positive	5674	(90.2)	986	(88.0)	0.038	83	(9.2)	83	(9.2)	n.s.
negative	613	(9.8)	135	(12.0)		818	(90.8)	818	(90.8)	
[unknown]	125	[1.9]	32	[2.7]						
Chemotherapy					n.s.					n.s.
no	4581	(71.4)	855	(74.2)		660	(73.3)	660	(73.3)	
yes	1831	(28.6)	298	(25.8)		241	(26.7)	241	(26.7)	
Endocrine therapy					<0.001					n.s.
no	3076	(48.0)	651	(56.5)		485	(53.8)	485	(53.8)	
yes	3336	(52.0)	502	(43.5)		416	(46.2)	416	(46.2)	

* Percentages of the presented subcategories are related to the sum of each item with available data; missing values are not taken into account. Hormone receptor positive: estrogen and/or progesterone positive (>1%). BCS: breast conserving surgery, RT: radiotherapy, n.s.: not significant.

**Table 2:** Cumulative incidence of local recurrences (LR) and lymph node recurrences (LNR) and Kaplan-Meier estimates of distant recurrence-free survival (DRFS) and overall survival (OS) for patients of the different cohorts. BCS + RT: breast-conserving surgery with postoperative radiotherapy; Mastectomy: mastectomy without radiotherapy; y: years.

Outcome	Treatment Modality	Entire Cohort			Case Control Cohort		
		Diagnosis 1998–2014			Diagnosis 1998–2014		
		7565 Patients			1802 Patients		
		5 y (%)	10 y (%)	*p*	5 y (%)	10 y (%)	*p*
LR				<0.001			0.025
	BCS + RT	3.2	8.2		4.6	9.4	
	Mastectomy	5.0	12.6		4.8	12.9	
							
LNR				<0.001			<0.001
	BCS + RT	0.9	2.2		0.7	2.0	
	Mastectomy	2.6	5.7		2.5	5.8	
							
DRFS				<0.001			0.013
	BCS + RT	94.5	90.2		93.8	89.4	
	Mastectomy	92.0	84.8		93.1	85.5	
							
OS				<0.001			<0.001
	BCS + RT	95.2	86.7		93.8	85.3	
	Mastectomy	90.5	77.6		92.2	79.3	

**Table 3:** Multivariable Cox regression analysis for local recurrence free survival.

Variable	Entire Cohort (*n* = 7565) Local Recurrence Free Survival			Case Control Cohort (*n* = 1802) Local Recurrence Free Survival		
	Hazard Ratio HR	95% CI	*p*-Value	Hazard Ratio HR	95% CI	*p*-Value
Local therapy			<0.001			0.013
BCS + RT	1			1		
Mastectomy	1.476	1.164-1.872		1.517	1.092–2.108	
Age at diagnosis			<0.001			<0.001
<40 years	1			1		
40–49 years	0.931	0.671–1.291		0.802	0.475–1.353	
50–59 years	0.521	0.370–0.732		0.309	0.172–0.554	
60–69 years	0.393	0.274–0.565		0.360	0.199–0.651	
≥70 years	0.357	0.228–0.561		0.168	0.075–0.379	
Tumour stage			<0.001			0.020
T1N0	1			1		
T2N0	1.177	0.899–1.541		0.916	0.584–1.434	
T1N1	1.147	0.855–1.538		1.014	0.601–1.712	
T2N1	2.091	1.565–2.795		1.969	1.204–3.220	
Resection status			0.604			0.330
R0	1			1		
R1/R2	0.808	0.360–1.812		1.773	0.560–5.618	
Grade			<0.001			0.320
G1	1			1		
G2	2.063	1.438–2.959		1.719	0.821–3.599	
G3/4	2.415	1.619–3.601		1.526	0.676–3.444	
Hormone receptor			0.012			0.104
positive	1			1		
negative	1.466	1.087–1.975		1.575	0.911–2.721	
Chemotherapy			0.402			0.462
yes	1			1		
no	1.110	0.870–1.417		1.172	0.768–1.789	
Endocrine therapy			0.382			0.955
yes	1			1		
no	0.808	0.360–1.812		1.010	0.706–1.447	

**Table 4:** Multivariable Cox regression analysis for lymph node recurrence-free survival (LNRFS).

Variable	Entire Cohort (*n* = 7565) Lymph Node Recurrence-Free Survival (LNRFS)			Case Control Cohort (*n* = 1802) Lymph Node Recurrence-Free Survival (LNRFS)		
	Hazard Ratio HR	95% CI	*p*-Value	Hazard Ratio HR	95% CI	*p*-Value
Local therapy			<0.001			0.013
BCS + RT	1			1		
Mastectomy	2.442	1.675–3.560		1.517	1.092–2.108	
Age at diagnosis			0.025			0.030
<40 years	1			1		
40–49 years	1.795	0.857–3.762		1.758	0.576–5.361	
50–59 years	1.143	0.539–2.423		0.715	0.215–2.376	
60–69 years	1.399	0.661–2.960		0.871	0.262–2.890	
≥70 years	0.603	0.238–1.526		0.286	0.058–1.411	
Tumor stage			0.006			0.331
T1N0	1			1		
T2N0	1.754	1.130–2.724		1.175	0.535–2.584	
T1N1	1.274	0.749–2.168		1.433	0.593–3.463	
T2N1	2.300	1.383–3.825		2.186	0.931–5.134	
Resection status			0.366			
R0	1			1		
R1/R2	0.403	0.056–2.888		NA *		
Grade			<0.001			0.082
G1	1			1		
G2	1.451	0.755–2.787		1.121	0.327–3.840	
G3	3.651	1.841–7.242		2.284	0.623–8.371	
Hormone receptor			0.120			0.973
positive	1			1		
negative	1.523	0.897–2.586		0.982	0.342–2.819	
Chemotherapy			0.221			0.593
yes	1			1		
no	1.303	0.853–1.990		1.223	0.585–2.557	
Endocrine therapy			0.193			0.702
yes	1			1		
no	0.770	0.520–1.141		0.885	0.475–1.652	

* NA: not applicable, HR not estimable because no event in the R1/2 group.

**Table 5:** Multivariable Cox regression analysis for distant metastasis free survival.

Variable	Entire Cohort (*n* = 7565) Distant Metastasis Free Survival (DMFS)			Case Control Cohort (*n* = 1802) Distant Metastasis Free Survival (DMFS)		
	Hazard Ratio HR	95% CI	*p*-Value	Hazard Ratio HR	95% CI	*p*-Value
Local therapy			0.044			0.008
BCS + RT	1			1		
Mastectomy	1.257	1.006–1.570		1.537	1.121–2.107	
Age at diagnosis			0.677			0.053
<40 years	1			1		
40–49 years	0.860	0.608–1.216		0.600	0.351–1.027	
50–59 years	0.826	0.592–1.153		0.497	0.292–0.845	
60–69 years	0.785	0.556–1.106		0.437	0.246–0.777	
≥70 years	0.891	0.601–1.321		0.592	0.314–1.118	
Tumor stage			<0.001			0.001
T1N0	1			1		
T2N0	1.895	1.489–2.411		1.258	0.820–1.932	
T1N1	1.577	1.196–2.080		1.520	0.933–2.477	
T2N1	3.755	2.930–4.812		2.516	1.608–3.936	
Resection status			0.209			0.587
R0	1			1		
R1/R2	1.445	0.813–2.568		1.377	0.435–4.364	
Grade			<0.001			
G1	0.215	0.141–0.327		NA *		
G2	0.514	0.421–0.629		NA		
G3	1			1		
Hormone receptor			0.050			0.706
positive	1			1		
negative	1.327	1.000–2.586		1.110	0.646–1.907	
Chemotherapy			0.656			0.517
yes	1			1		
no	0.951	0.762–1.186		0.874	0.583–1.312	
Endocrine therapy			0.013			0.174
yes	1			1		
no	0.770	0.627–0.946		0.782	0.549–1.114	

* NA: not applicable, HR not estimable because no event in the R1/2 group.

**Table 6:** Multivariable Cox regression analysis for overall survival.

Variable	Entire Cohort (*n* = 7565) Overall Survival (OS)			Case Control Cohort (*n* = 1802) Overall Survival (OS)		
	Hazard Ratio HR	95% CI	*p*-Value	Hazard Ratio HR	95% CI	*p*-Value
Local therapy			0.011			0.004
BCS + RT	1			1		
Mastectomy	1.268	1.055–1.525		1.452	1.124–1.875	
Age at diagnosis			<0.001			<0.001
<40 years	1			1		
40–49 years	1.011	0.674–1.517		0.439	0.240–0.804	
50–59 years	1.273	0.870–1.861		0.599	0.346–1.038	
60–69 years	1.757	1.203–2.565		0.854	0.494–1.476	
≥70 years	4.552	3.089–6.710		2.335	1.342–4.065	
Tumor stage			<0.001			<0.001
T1N0	1			1		
T2N0	1.763	1.446–2.150		1.633	1.175–2.270	
T1N1	1.529	1.214–1.925		1.375	0.887–2.130	
T2N1	2.892	2.337–3.580		2.589	1.786–3.753	
Resection status			0.608			0.712
R0	1			1		
R1/R2	1.144	0.685–1.911		1.184	0.484–2.896	
Grade			<0.001			0.033
G1	1			1		
G2	1.406	1.100–1.798		1.968	1.028–3.768	
G3	2.165	1.645–2.848		2.432	1.227–4.820	
Hormone receptor			0.076			0.606
positive	1			1		
negative	1.254	0.986–1.612		1.135	0.702–1.834	
Chemotherapy			0.481			0.708
yes	1			1		
no	1.075	0.880–1.313		1.075	0.736–1.570	
Endocrine therapy			0.662			0.709
yes	1			1		
no	1.039	0.876–1.232		0.946	0.708–1.26	
